# Integration of Membrane-Based Pretreatment Methods with Pressure-Retarded Osmosis for Performance Enhancement: A Review

**DOI:** 10.3390/ma18051020

**Published:** 2025-02-26

**Authors:** Sara Pakdaman, Giti Nouri, Catherine N. Mulligan, Fuzhan Nasiri

**Affiliations:** Department of Building, Civil and Environmental Engineering, Concordia University, 1455 de Maisonneuve Blvd. W., Montreal, QC H3G 1M8, Canada; sara.pakdaman@mail.concordia.ca (S.P.); giti.nouri@concordia.ca (G.N.); fuzhan.nasiri@concordia.ca (F.N.)

**Keywords:** pressure-retarded osmosis, wastewater pretreatment, membrane modification, fouling mitigation, renewable energy, hybrid systems

## Abstract

Osmotic energy provides an emerging renewable alternative by leveraging the salinity gradient between two solutions. Among these technologies, pressure-retarded osmosis (PRO) has attracted attention; however, its deployment is hindered by obstacles resulting from impurities in feed and draw solutions and lack of suitable membranes. This review explores the integration of membrane-based pretreatments with PRO, highlighting their influence on resolving the technical drawbacks of standalone PRO systems. Membrane-based pretreatments have shown considerable potential to overcome these challenges by improving the quality of water, reducing membrane fouling and enhancing its performance, and ultimately contributing to recovery of energy, resulting in higher power density. Additionally, the use of different nanomaterials has been proposed for membrane modification to optimize PRO performance. Moreover, the study investigates recent advancements in hybrid configurations for harnessing existing infrastructure and to enhance energy efficiency. Offering a comprehensive review on this integrated approach contributes to valuable insights for advancing membrane-based hybrid systems toward commercial viability. Consequently, investment in developing advanced computational modeling and experimental validation, utilization of advanced membrane materials with higher fouling resistance, and optimization of system configurations by using dual-stage and multi-stage designs are required to overcome these limitations.

## 1. Introduction

**Figure 1:**
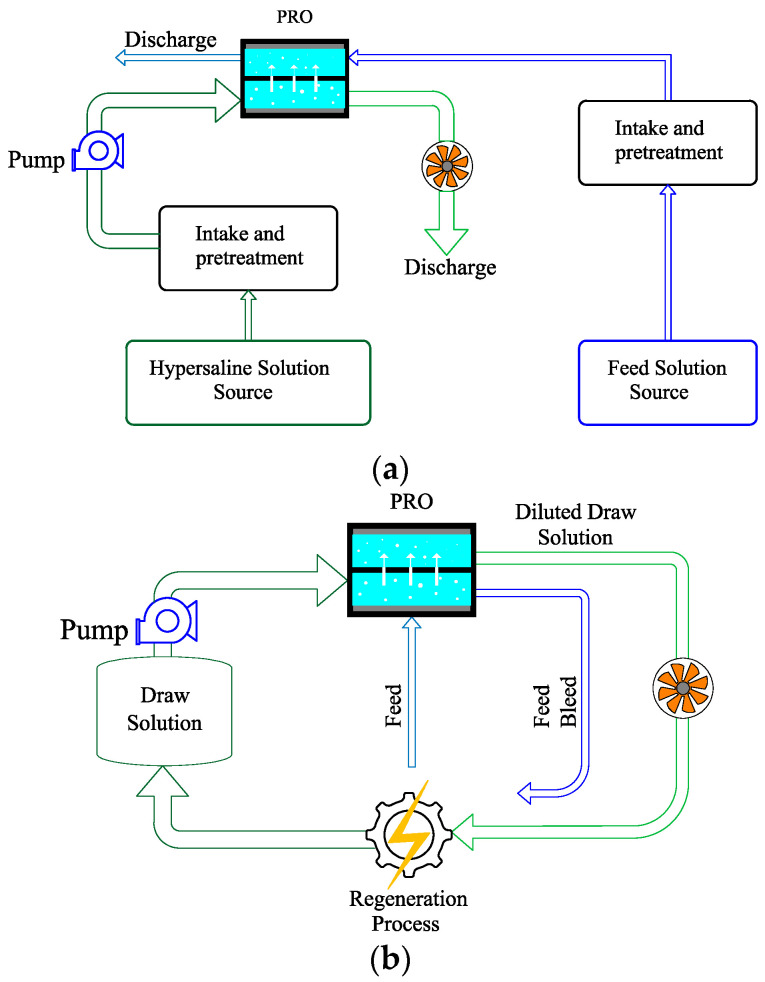
Configurations of PRO: (**a**) open loop and (**b**) closed loop (adapted from Zadeh et al. [1]).

There is an urgent need for novel, cost efficient, and environmentally friendly energy technologies to address the globally rising energy demands and concerns on greenhouse gas emissions contributing to climate change. Additionally, the depletion of the current conventional sources of energy and their associated greenhouse gas emissions emphasize the urgency of this need. Salinity gradient energy, also known as osmotic or blue energy, is a kind of renewable energy that utilizes the difference in salinity levels in two solutions to derive energy and pressure-retarded osmosis (PRO) has attracted attention as one of the most emerging technologies in this domain. A wide range of feed solutions and draw solutions (FSs and DSs) can be employed in PRO such as freshwater, river water, seawater, effluents, and concentrated brines. PRO has two configurations, including the open-loop and closed-loop PRO systems. As depicted in Figure 1, in an open-loop PRO, fresh and saline waters are combined. Then, both streams are discharged after the osmosis process. The open-loop PRO system incurs higher expenses due to the treatment of feed and draw solutions and the required energy for stream transportation. Therefore, the geographical location of these plants limits their applicability to areas near inlet streams. Additionally, membrane fouling is increased in this system, leading to higher costs of membrane maintenance. In contrast, in a closed-loop PRO system, streams leaving the membrane are regenerated and recirculated into the system, making it environmentally advantageous since it mitigates the discharge need. Closed-loop PRO provides the benefit of selecting the draw solution with greater precision, allowing for customization based on the energy resources at specific areas, making them more flexible, practical, and efficient [1].

Utilizing these water solutions can lead to some challenges like internal and external concentration polarization (ICP and ECP), membrane fouling, and reverse salt flux (RSF), hindering its practical implementation [2,3,4]. Membrane fouling happens when suspended solids, organic materials, microbes, etc. are deposited on the surface or within of the membrane, causing a reduction in permeate flux [5]. Fouling is considered reversible when foulants are deposited on the membrane surface, while it is irreversible when they are deposited inside membrane pores. Membrane fouling hinders the movement of permeates affecting its performance. Consequently, to ensure passage of permeates through the membrane, higher pressure than normal is required [6]. This leads to high consumption of energy, more downtime, and reduction in membrane area. There are several forms of fouling, including colloidal, organic, inorganic, and biofouling [7]. It is worth noting that membrane fouling relies on feed properties, such as pH and ionic strength and characteristics of the membrane like roughness, hydrophobicity, etc., and conditions of the process including crossflow velocity, transmembrane pressure and temperature [8,9].

Therefore, pretreatment of feedwater, which is classified as physical and chemical, is essential for removing impurities and mitigating the effects of these limitations [5]. The effect of feedwater composition emerges as an important factor in the effectiveness of pretreatment. Therefore, an appropriate pretreatment method should be selected considering the composition of feedwater and the specific operational conditions of each application to make membrane separation more effective [10]. It plays a vital role in the success of membrane processes by enhancing the characteristics of feed flow, reducing membrane fouling, maintaining the constant flux, and contributing to utilization of energy, resulting in higher power density and lifespan of the membrane [11,12,13]. Among several methods, membrane-based pretreatments are promising alternatives to address PRO’s challenges through reducing the presence of impurities and foulants in FS and DS, thus, leading to membrane performance enhancement along with operational efficiency. Membrane-based pretreatments make reclamation of water from wastewater a good option [14,15,16,17]. Furthermore, integrating these pretreatments with the PRO process enables the effective usage of byproducts with high salinities, improves energy recovery, and diminishes discharge associated environmental impacts [18]. In hybrid membrane-based pretreatments, one or more membrane techniques are combined to give a better performance than either of the methods as a standalone treatment [19]. Therefore, the drawbacks of each process are complemented by the other one, thereby improving the production of treated water with high quality [20].

This review paper aims to explore the interrelations between the PRO process and membrane-based pretreatments and how this integration contributes to overcoming the limitations of a standalone PRO system. It uniquely addresses a new and distinctive insight into the integration of membrane-based pretreatments with PRO, which has not been investigated in previous literature reviews. This study focuses on the role of pretreatments in refining the quality of feedwater entering PRO, improving the performance of the membrane by modifying it with different materials, enhancing the recovery of energy, and consequently leading to higher operational efficiency and less environmental impacts. Moreover, this review identifies the gaps and limitations in the current studies and suggests strategies to address these challenges and develop an advanced and practical hybrid system.

## 2. Fundamentals of Pressure-Retarded Osmosis

### 2.1. Principles and Mechanisms

**Figure 2:**
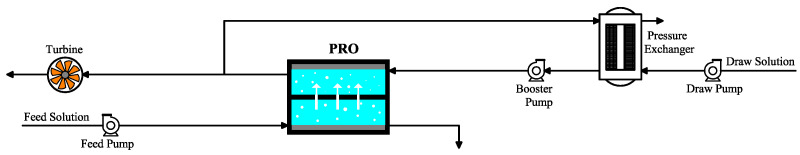
A typical PRO process (adapted from Bajraktari et al. [22]).

PRO harnesses the osmotic energy that stems from the difference in salinities between two solutions. The water goes from a feed solution side with low salt concentration toward a draw solution side with high concentration through a semi-permeable membrane, against a hydraulic pressure [21]. The accumulated water in the draw side runs through a turbine and generates energy [3]. Figure 2 depicts a typical PRO process [7].

The membrane is the core part of PRO that determines the overall performance of a PRO process. There are two types of membranes applied in PRO: a thin-film composite (TFC) and cellulose acetate (CA) or cellulose triacetate (CTA). CTA membranes have been used widely in PRO because of several advantages, like high hydrophilicity, which improves water flux and mitigates membrane fouling, as well as offers higher mechanical strength and tolerance to chlorine. Their hydrophilic nature is beneficial in the PRO process since wetting the membrane increases the water flux and decreases ICP. However, the major drawback of these membranes is their limited tolerance to the change in pH. In TFC membranes, layers of dissimilar materials are merged to form a single membrane. This specification permits the utilization of material combinations to optimize membrane performance and durability. These membranes are characterized by a wide range of feed pH unlike CTA membranes, while they have less tolerance to oxidants and chlorine [23]. TFC membranes show higher water flux and efficiency because of their greater flexibility in tailoring their structural and functional characteristics leading to enhanced performance of support and selective layer [2]. When the TFC membrane was first reported, it had higher water permeability (~0.74 L m^−2^ h^−1^ bar^−1^) than cellulose-based membranes [24]. A water flux of 57 L m^−2^ h^−1^ was achieved using an optimized TFC membrane, which was among the highest water fluxes [25]. The TFC membrane demonstrated higher power density compared to CTA membranes. The maximum power densities for the CTA and TFC membranes were reported as 7.7 W/m^2^ and 10.6 W/m^2^, respectively [23].

### 2.2. Water Flux and Power Density

Water flux ($J_{w}$), the permeation of water through a membrane, can be calculated by the following equation:

(1) $$J_{w}=A\left(∆\pi -∆P\right)=A(\pi_{Draw}-\pi_{Feed}-∆P)$$

where A is the water permeability coefficient and ∆π and ∆P are the difference in osmotic pressure and the transmembrane pressure, respectively. $\pi_{Draw}$ and $\pi_{Feed}$ represent the bulk osmotic pressures of draw and feed solutions, respectively. In this equation, ideal conditions (perfect selectivity of membrane and optimal hydrodynamics in the draw and feed channels) are assumed, expressing that the concentrations at the surface of the membrane and the bulk concentrations are equivalent [23]. Obtaining a high-power density (W/m^2^) is essential for PRO to be economically viable and can be obtained using the following relationships:

(2) $$W=J_{w}\;\left(∆P\right)$$

(3) $$J_{w}=-D_{w}\frac{{dc}_{w}}{dx}$$

where w denotes the power density, while $D_{w}$ and $c_{w}$ are water’s diffusion coefficient and its concentration, respectively, and *x* is the coordinate to the perpendicular surface of the membrane. When ideal mixing is assumed, the maximum w can be achieved when the hydraulic pressure is half the theoretical osmotic pressure [18].

(4) $$W_{max}=A\frac{{∆\pi }^{2}}{4}$$

### 2.3. Historical Development

In 1973, Loeb first introduced PRO, by conducting feasibility studies on generating energy from saline water. Later in 1975, Loeb and Norman patented a PRO-based heat engine and formally suggested the term PRO. Despite this, it did not attract attention until the 2000s, which led to ongoing academic and industrial developments for commercialization. In 2009, the Statkraft project was a pilot plant implementation of PRO for the production of 10 kW salinity gradient energy from river water and seawater. However, only 5 kW was achieved due to membrane performance associated limitations. Following this, notable partnerships like the collaborations between Statkraft and Hydro-Quebec, as well as Nitto Denko and Hydranautics, emerged. However, PRO’s commercialization remained challenging due to thermodynamic barriers of extracting energy from mixing river water and seawater, insufficient spacer and module designs, and high fouling propensity, hindering its performance optimization. Efforts of advancing PRO continues. For example, Japan’s Mega-ton water project and Korea’s Global Membrane Value Partnership project were initiated to enhance the efficiency of energy production and reduce environmental impacts by focusing on hybridizing PRO with other desalination technologies. These integrations aim to address the limitations of standalone PRO systems and enable more sustainable and efficient applications of salinity gradient energy [2,22,26,27].

### 2.4. Challenges and Limitations

The maximum power density of PRO is linked to the pure water permeation across the membrane. However, in practice, the actual achieved power density falls short of theoretical predictions since there are several factors, including ICP, ECP, RSF, and membrane fouling contributing to the loss of effective osmotic pressure. ICP is the increase in the salt concentration at the interface originating from the accumulation of solutes within the membrane porous support layer. This phenomenon reduces the performance of PRO and power density, especially when combined by the structural properties of the membrane. Membranes with optimized designs capable of balancing permeability and mechanical strength are required to address ICP. On the other hand, ECP occurs when solutes accumulate at the active layer-solution interface at the external surface of the membrane that imposes significant constraints on the efficiency of the system at higher water fluxes. Additionally, as the PRO process continues, RSF occurs by the accumulation of salts from draw side in the feed side, which further reduces the effective pressure difference across the membrane under pressurized conditions contributing to ICP [2,18].

## 3. Innovations for Improving Membrane Performance

TFC is a kind of semipermeable membrane which consists of at least two layers and can be prepared with different techniques including nonsolvent-induced phase separation that is usually used for preparation of the membrane support layer. Then, the active layer is formed on the top surface of the membrane via interfacial polymerization. TFC is utilized generally throughout the PRO process due to its durability and higher tolerance towards various pH conditions of the feed solution [28]. However, TFC membranes are often faced with problems including, fouling, CP, high RSF, and low salt rejection. Moreover, challenges cause inefficiency in desalination and other membrane-based separation technologies. Thus, advancement of the membrane and optimizing the membrane water permeability and its structural parameters are important factors that can lead to better membrane performance [29].

The integration of nanoparticles into TFC membrane polymer solutions (support layer) or dispersion on its active layer lead to the production of thin-film nanocomposite (TFN) membranes. It is a technique that greatly enhances the membrane selectivity by enhancement of the water permeability and reduction of salt permeability [30]. In addition to separation properties of the membranes, their durability and stability towards harsh chemicals and thermal conditions can be increased with higher hydrophilicity and mechanical strength, which increases the generation of sustainable energy within the osmotic-driven process [31]. As a result, adding different materials can improve the membrane structural parameters and other essential characteristics.

### 3.1. Modification of the Active Layer

The goal for improvement of membrane-based technology has been to modify and prepare membranes with better properties. Different types of membranes, including TFC, flat sheet, hollow fiber, and membranes with nanomaterials known as TFN membranes, have been utilized in membrane-based technologies. Researchers are still working to increase the performance of the membranes that can directly impact membrane technology. Changing the membrane surface is a new method to address fouling and enhance membrane performance. In recent studies, TFC membranes obtained great attention due to high ion selectivity and water permeability that are the most important factors in osmotic-driven processes [32]. Recent developments in osmotic membrane technology, distinguished by their exceptional structural and transport properties, include incorporating functionalized nanoscale materials that have potential for producing highly efficient TFN membranes [29]. Specifically, for generation of osmotic power within PRO, membranes should have a combination of forward osmosis (FO) and reverse osmosis (RO) membranes properties. The RO membrane has high salt rejection, low water flux, and high mechanical strength due to its thickness. It is not suitable for PRO application due to its low water flux that leads to low power density [33]. For example, membrane thickness is a critical factor, which influences the results, including water flux and power density. As mentioned before, TFC is made of a dense active layer and a porous support layer. The higher thickness of these layers affects the presence of ICP, which can significantly reduce the osmotic driving forces [34]. Generally, membranes with low structural parameters, which is the relationship between thickness, tortuosity, and porosity, are preferred, to minimize the ICP [27]. Consequently, membrane thickness needs to be optimized to control the ICP and increase the water flux and power density, while withstanding the high hydraulic pressure that is applied during the PRO process, which is less than the applied pressure throughout the RO process.

**Figure 3:**
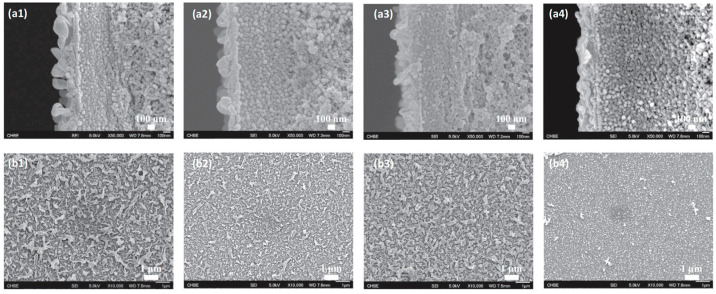
(**a**) Cross-section and (**b**) top surface FESEM images of modified membranes with Na-CQD-9. (**a1**,**b1**) TFC membrane and (**a2**,**b2**) TFN with 0.5 wt.% of Na-CQD-9. (**a3**,**b3**) TFN with 1 wt.% of Na-CQD-9. (**a4**,**b4**) TFN with 2 wt.% of Na-CQD-9 (“Reprinted from Journal of Membrane Science, Vol. 551, Wenxiao Gai, Die Ling Zhao, Tai-Shung Chung, Novel thin film composite hollow fiber membranes incorporated with carbon quantum dots for osmotic power generation, Pages 94–102, Copyright (2018), with permission from Elsevier.” [36]).

On the other side, the FO membrane has closer properties including its porosity, thickness, and water flux to use in the PRO system. However, the FO membrane does not have enough mechanical strength and will be deformed under the applied hydraulic pressure during the PRO process [35]. Since PRO is still suffering from a lack of membranes with high performance, researchers have been working on membrane with high water permeability and mechanical strength. In a study, sodium-functionalized carbon quantum dots (Na-CQDs) were incorporated into a polyamide layer of TFC hollow fiber membrane. Figure 3 shows the morphology of modified membranes with different wt.% (0.5, 1, 2) of Na-CQD-9 and TFC membranes with field emission scanning electron microscopy (FESEM). According to these images, membranes with 0.5 and 1 wt.% of Na-CQD-9 exhibit a denser polyamide layer with reduced migration of aqueous solution to the organic phase attributed to the lower concentration of the additive. Moreover, 2 wt.% of the additive displays a looser polyamide selective layer and higher migration of aqueous solution to the organic phase due to a higher concentration of the additive. By analyzing the membranes during the PRO process, it was found that 1 wt.% of Na-CQD-9 is the optimal amount to increase to water flux and power density to 53.54 L m^−2^ h^−1^ and 34.2 W/m^2^ at 23 bar, which is higher than an unmodified TFC membrane [36].

In another study [37], a hydrophobic porous polymer was modified with a hydrophilic sulfonate group to make PP-SO_3_H. Then, this material was incorporated to the polyamide layer of the TFN membrane. The properties of the membrane surface including its hydrophilicity and porosity were improved and led to an enhancement of water flux with acceptable salt rejection. The best performance of the membrane was achieved with 0.002 wt.% of PP-SO_3_H. Water flux reached 46.3 L m^−2^ h^−1^, which is higher than the water flux of the membrane without any modification (31.4 L m^−2^ h^−1^). 1.0 M NaCl and deionized water were used as a draw and feed solution, respectively. Furthermore, 14.6 W/m^2^ power was generated at a 17 bar as hydraulic pressure, which is more with the unmodified membrane [37]. Many studies have shown that forming an interlayer between the porous support layer and polyamide layer is a preferred strategy for making high-performance TFC membranes. Han et al. [38] demonstrated that incorporating polydopamine (PDA) can enhance membrane hydrophilicity and form a smother surface for better formation of the polyamide layer. Water flux reached 24 L m^−2^ h^−1^, making this membrane suitable for water desalination and FO application with low salt leakage [38].

The membrane performance during the osmosis process can be influenced by temperature. By increasing the temperature, the water flux and reverse salt flux of the membrane will increase. Passing more salts through the membrane has a negative impact on overall performance of the membrane and osmosis process [39]. By modification of the surface of the membrane, it is possible to increase its compatibility with a high temperature water. When graphene oxide (GO)/PDA was coated on the polyethersulfone support layer, it modified the active layer with a different wt.% of triaminopyrimidine (TAP) into the m-phenylenediamine monomer. GO and PDA were utilized to make an interlayer to enhance the membrane’s water permeability and thermal resistance. The interfacial polymerization process on a PDA/GO interlayer ensured more unity diffusion of the aqueous solution with a thinner, smoother selective layer. Based on the results, water permeability of the membrane with TAP reached 0.33 L m^−2^ h^−1^ bar^−1^ compared to 0.24 L m^−2^ h^−1^ bar^−1^ without it. Additionally, the modified membrane with higher concentrations of TAP increased the glass transition temperature to above the 80 °C because of the greater cross-linking density, which makes the polymer chains more rigid. In addition, the TAP-modified membrane had higher decomposition temperatures between the range of 450–550 °C, with 2 wt.% of TAP showing the best thermal performance and resistance compared with the unmodified membrane [40]. In another study [41], polyacrylonitrile (PAN) was used as the polymer in the doping solution and different wt.% of biowaste-derived nitrogen-doped carbon quantum dots were added to the polyamide layer. The results showed that 0.5 wt.% of this additive enhanced water flux to 24.6 L m^−2^ h^−1^, which is approximately 55% more than the unmodified membrane, and it significantly reduced the reverse salt flux to 3.8 gMH, which is 43% lower than the TFC membrane without any additives [41].

### 3.2. Modification of the Support Layer

Over the past decades, many notable advancements and changes have been seen in membrane technology by adding various types of nanomaterials including GO, zeolite, titanium dioxide (TiO_2_) and others into the support layer of the membrane [42]. In a study [43], researchers used GO to enhance the membrane power density, hydrophilicity, structural parameters, mechanical strength, and resistance toward fouling. The remarkable properties of GO, including its two-dimensional single-layer nanosheets and hydrophilic functional groups, improved the antifouling tendency and hydrophilicity of the membranes. They added a different wt.% of GO into the dope solution. As draw and feed solutions, 1 M NaCl and DI water were used, respectively. Among modified membranes, 0.25 wt.% of GO had excellent water flux, RSF, and high porosity compared with unmodified membrane with the highest power density around 8.36 W/m^2^ at more than 15 bar pressure [43].

As discussed, previously, nanofiltration has emerged as a leading membrane-based technology for wastewater treatment due to its low energy consumption. A study investigated the modification support layer of polysulfone with different wt.% of a zeolite nanomaterial in nanofiltration (NF) application. The results showed that 0.1 wt.% of zeolite increased water flux up to 17 L m^−2^ h^−1^, and there was high salt rejection of MgSO_4_ and Na_2_SO_4_ [44]. Zeolite was used in another study [45] for PRO and FO applications to increase the water permeability and mitigate internal concentration polarization. They synthesized membranes through phase inversion with 0–0.6% zeolite added to polyethersulfone. By characterizing the membrane, they realized by using zeolite the surface porosity increased. In addition, using zeolite optimized the enhancement of water flux compared with the membrane without it during the PRO process. Moreover, membranes with 0.4 wt.% zeolite showed a high salt rejection (94.7%), with higher water flux in comparison with TFC without any modifications. As a result, zeolite can effectively increase the water flux, decreasing the structural parameters that lead to the reduction in internal concentration polarization during osmotic processes [45].

NF and commercial TFC membranes are not appropriate for FO and PRO processes due to severe ICP. In a research study [46], researchers used treated palm oil mill effluent and 4 M MgCl_2_ as feed and draw solutions, respectively. They prepared membranes with different weight ratios of polyphenylsulfone (PPSU) and PAN (PPSU/PAN). It was found that the 1:5 ratio of PPSU/PAN with PDA and 0.5 mg/L GO coated on membrane surfaces enhanced 41% and 67% of water flux in PRO and FO processes, which was better than commercial NF membrane performance [46].

In another study [47], researchers developed a dual-layered nanocomposite membrane with 0.25 wt.% of GO and a thin polyamide layer on the top layer and different wt.% of halloysite nanotubes on the bottom surface. The prepared membranes demonstrated favorable membrane characteristics, including high porosity, open-bottom surface, appropriate top-skin surface morphology for forming the active layer, and high mechanical strength, which are crucial for achieving optimal performance of PRO. The results show that halloysite nanotube loading of 4 wt.% provides a high power density of 16.7 W/m^2^ and specific reverse solute flux of about 2.4 g/L at 21 bars when using 1 M NaCl as the draw solution and deionized water as the feed solution, which are better than a commercial membrane used in the PRO system [47]. GO was used in another study [48] and embedded with polyethersulfone support layer for preparation of a hollow fiber membrane. As draw and feed solutions, 1 M NaCl and deionized water were used, respectively, in the PRO process. Ultimately, 0.2 wt.% of GO caused the highest water flux (43.74 L m^−2^ h^−1^) and power density (14.6 W/m^2^) at 16.5 bar, which are higher than the properties of the membranes without additives [48]. Table 1 shows the summary of literature on membrane active and support layer modification and indicates that of all nanomaterials discussed in this study, the paper that used Na-CQDs has the highest ability to generate osmotic power with 34.20 W/m^2^. CQDs have low toxicity, high hydrophilicity, and are environmentally friendly and affordable compared with other nanomaterials. For example, in contrast to GO, CQDs can easily be incorporated into various membranes including NF, RO, and PRO. Moreover, their small particle size and abundance of functional groups allow them to form chemical bonds with a wide variety of compounds [36,41,48].

## 4. Role of Nanomaterials in Development of Anti-Fouling Properties of Membranes

Membrane fouling is a common problem in desalination and water treatment systems. The accumulation of suspended organic materials on the membrane surface or inside its structure leads to membrane fouling. Inorganic minerals like calcium, magnesium, sulfate, and colloidal particles deposit onto the membrane, leading to membrane scaling [49]. Consequently, the fouling will raise the membrane capital costs, which will have an impact on the membrane-based technologies for water treatment. Fouling and scaling may cause the membrane surface to develop a cake layer, blocking the pores of the membrane [50,51]. Water flux and membrane lifespan can be decreased by fouling of the membrane. Pretreatment is an effective way to mitigate this problem that will be discussed. However, another option is chemical modification of the membrane or incorporation of different nanomaterials to change the separation properties and control the interaction of membrane with foulants.

A study [52] focused on developing a FO membrane by modifying polysulfone with a polyamide membrane and zwitterionic arginine by using a mild amidation reaction to enhance membrane hydrophilicity, antifouling, and antibacterial properties. Membrane properties analyzed after treating oily wastewater, including antifouling tests with emulsified oils and antibacterial tests against *Escherichia coli*, showed a water flux increase of 113.2% and comparable salt rejection with an unmodified membrane. Moreover, prepared membranes exhibited high antifouling capacity with flux recovery ratios after washing, with 92%, 87%, and 86% for cationic, neutral, and anionic emulsified oils, respectively. Regarding the membrane antibacterial efficiency, it showed approximately 96% antibacterial properties against *Escherichia coli*. Based on the results, they concluded that this nanomaterial is practical for treatment of actual oily wastewater and is scalable due to its superior antifouling properties [52]. Different weight percentages (0.01–0.1) of MoS_2_ and MoS_2_-Ag nanofillers were added to TFN membranes via interfacial polarization to mitigate organic fouling and enhance the water treatment and desalination efficiency. It was identified that the addition of Ag nanoparticles can increase the negative charge and hydrophilicity of the membrane surface in comparison with membranes with only MoS_2_, and it led to higher water flux and fouling resistance over a longer period of time. For example, they compared the water flux of the TFC, TFN with MoS_2_, and TFN with MoS_2_-Ag after 270 min and 540 min in the presence of organic foulants. It was identified that the TFC water flux decline was approximately 36% and 45%, while membranes with MoS_2_ had 17% and 31% and TFN with MoS_2_-Ag had 8% and 11% decline at 270 and 540 min, respectively. During the FO process, water flux of the TFN membrane with MoS_2_-Ag increased by approximately 35% (reached 16.9 L m^−2^ h^−1^ from 12.5 L m^−2^ h^−1^ for TFC without any additive), and significant fouling resistance was achieved with 0.02 wt.% of MoS_2_-Ag in comparison with TFC membranes [53]. Combination effects of GO and quorum quenching using *Pseudomonas* quinolone signal inhibitors on addressing biofouling of TFC membranes in FO and PRO processes was explored. The separation properties of the membranes indicated that membrane with GO has the higher water permeability, around 2.65 L m^−2^ h^−1^ bar^−1^ compared with TFC membrane with 1.82 L m^−2^ h^−1^ bar^−1^. Moreover, a methyl anthranilate inhibitor was applied to prevent biofilm formation. Results showed that methyl anthranilate reduced the substances of extracellular polymeric generation and thickness of biofilm by up to 49% and 47%, respectively, in FO and PRO processes. In addition, TFN with GO and addition of methyl anthranilate achieved 25% higher water flux and the highest resistance towards bacteria, which form a biofilm on the membrane surface compared with TFC without nanoparticles [54]. In another research study [55] centered around enhancing the antifouling activity of the membrane, functionalized nanodiamond (ND) particles were added onto the top surface of the membrane, resulting in water flux declining by 15% and 9% in the presence of sodium alginate and bovine serum albumin, while TFC membrane without any additives decreased by 42% and 21% in the presence of sodium alginate and bovine serum albumin, respectively. Moreover, *Escherichia coli* was used to investigate the antibacterial performance of membranes with ND particles. According to the colony plating test, they found that the membrane modified with ND inactivated 63% of the bacteria, while the TFC membrane inactivated only 1.3% [55]. Consequently, developing suitable membranes for desalination and wastewater treatment made it possible to show the feasibility of membrane-based technologies for wider-scale applications. Table 2 shows the summary of different nanomaterials that were used to enhance the fouling resistance of the membranes.

## 5. Membrane-Based Pretreatments

The world is facing a huge challenge of lack of safe and clean water. Water quantities are declining due to excessive use for domestic and industrial purposes. Moreover, mismanagement of industrial wastewater treatment has become a critical issue that needs to be mitigated. Membrane-based pretreatments are gaining attention because of their capability to remove a wide range of fouling agents. The effectiveness of these methods relies on the pore size of the membrane or molecular weight cut-off [56]. Membrane-based pretreatment methods require lower energy in comparison with other techniques by harnessing membranes with distinct physical and chemical characteristics to improve performance of PRO. Examples of such pretreatments include microfiltration (MF), ultrafiltration (UF), NF, membrane distillation (MD), RO, and FO [57].

There are differences between these techniques, including the pressure that is applied during the process and the pore size. These membranes can prevent solutes passing through the membrane based on their pore size. The pore size of this membranes is, 0.0001–0.001 µm for RO, 0.0005–0.01 µm for NF, UF is 0.005–0.5 µm, and 0.05–1.0 µm for MF membranes. Therefore, the smaller size particles remain behind these membranes and are not allowed to pass through the membrane [58]. Furthermore, the pressure that they can operate under is 10–100 bar, 5–40 bar, 1–10 bar, and <2 bar for RO, NF, UF, and MF, respectively. These membranes are efficient in removing organic materials, divalent ions, and some small particles. For example, some bacteria, large size colloids, and some kinds of macromolecules are removed by using ultrafiltration membranes because of their size [59]. In a study, researchers used Fe_3_O_4_@SiO_2_–CS biocompatible materials for synthesizing NF membranes and increasing their performance. The modified membranes showed high rejection rates of Na_2_SO_4_ and around 98% removal of the heavy metals Pb^2+^, Cu^2+^, and Cd^2+^. Moreover, 98.2% dye retention was achieved by all prepared NF membranes for both cationic and anionic dyes, including methylene blue, congo red, and reactive black 5 [60]. Different combinations of these pressure-driven membrane innovations have been applied in a range of wastewater treatment scenarios. They can also be used as a pretreatment for other unit strategies, and they can be integrated with other technologies to decrease the fouling problem. Furthermore, the wastewater of different industries can be treated to produce high quality water, and it can be reused in other industries, which is cost-effective. A study investigated the efficiency of NF and RO hybrid systems for treating a major industrial pollutant. During the process, significant removal of contaminants, including 99.80% of total dissolved solids and 99.99% of potassium, was obtained. This combination has effectively treated domestic and other wastewaters and produced water with high quality that is safe and environmentally friendly to release even with changing the input conditions [61].

Another study [62] explored the removal of oil, grease, and organic materials that are dissolved and suspended as solids from the oilfield-produced water after the bioremediation process by the MF, UF, and NF posttreatment methods. They compared the treated water with safe environmental disposal limits. Among MF, UF, and NF, the highest rejection was by NF, and it could remove multivalent ions including Mg^2+^, Pb^2+^, Sr^2+^, and Ca^2+^ [62]. The mining industry, especially gold mining, is very important from an economic point of view. It has numerous environmental effects due to this activity. High levels of heavy metals and acidic materials can be found in mining wastewater. However, membrane separation technologies, particularly NF and UF filtration, offer great promise for mining wastewater reusing and treatment [63]. Grossi et al. [64] studied gold mining wastewater. They investigated the UF and RO processes in both laboratory and pilot scales to treat a mining wastewater from the blasting stage, which was contaminated with nitrogen compounds. Aspects including recovery of the water fraction and transmembrane pressures were examined in terms of operational ability and economic impact. The observations showed that UF-MF pretreatment was essential for decreasing the inclination to fouling, and osmotic pressure did not play a key role in the overall performance, unlike other gold mining effluents. Therefore, the higher water recovery fractions were achieved while still maintaining a satisfactory permeate flux. From a technological and financial aspect, the system has shown itself to be a viable substitute, with the potential to save about 367,000 m^3^/year of freshwater [64]. Amarala et al. [65] evaluated the performance of a UF, NF, and RO integrated system to treat a gold mining wastewater from the pressure-oxidation stage for recovery of water and acid. They effectively produced high-quality water and recovered acid for reuse in ore processing [65].

### 5.1. PRO Integrated with MF, UF, and NF

To evaluate the effect of pretreatment on the performance of PRO, it is necessary to analyze the correlation between the quality of water, decline of flux, power density, and the predicted rate of membrane fouling prior to process operation. To address these aspects, Choi et al. [66] conducted a comprehensive experimental study on MF pretreatment using various FSs with different compositions. They evaluated the presence of particulate matter in the feedwater by measuring turbidity and total dissolved solids and quantified organic matter via conductivity, ultraviolet absorbance at 254 nm, and total organic carbon. To predict behavior of fouling based on feedwater properties, silt density and the modified fouling index were utilized. The results indicated that MF reduced turbidity substantially, while there were only minor changes in other parameters. The obtained power density was higher when pretreated waters were employed, highlighting the influence of MF on performance due to a reduction in the cake layer concentration polarization [66]. In another study [67], UF pretreatment was examined in a PRO system using excitation-emission matrix spectroscopy, and a decline of a 30% in permeate flux without chemical additives was demonstrated. In contrast, a flux decline was mitigated when UF was combined with a chemical additive, enabling continuous 15-day operation of the system [67]. Membrane fouling in PRO involves complex interplays with inorganic scaling, which is beyond organic components.

Abbasi-Garravand et al. [68] compared UF with traditional multi-stage flash pretreatment. The findings revealed that UF performed better than MSF in removing foulants by representing less permeate flux reduction of 3% compared to that of 52% for FS pretreated with MSF. Therefore, the power density remained more stable when UF was utilized as the pretreatment over time [16]. Chen et al. [69] performed research to examine the performance of PRO by assessing membrane fouling when three NF membranes were employed to pretreat RO brine from wastewater. The FS had a high concentration of organic compounds and divalent ions contributing to scaling. The results showed that NF successfully removed Ca^2+^, Mg^2+^, SO_4_^2−^, and PO_4_^3−^ ions, while the concentrations of silica remained unaffected. Since the pretreatment was effective in rejecting organic compounds, the power density increased from 4.4 to 13.5 W/m^2^ [69]. In another study [70], the application of UF, NF, and low-pressure reverse osmosis (LPRO) were explored for mitigating ICP, inorganic and organic scaling, and the accumulation of silica. It was indicated that UF was ineffective, while NF demonstrated superior effectiveness in removing most inorganic and organic compounds, except for silica. Additionally, NF maintained a stable flux, with only a 22.7% reduction over 24 h. LPRO yielded better water quality and stability than NF, which made it the most effective pretreatment owing to its near-complete removal of silica and inorganic ions [70].

Lee et al. [71] conducted a pilot scale study to evaluate the effectiveness of UF on the system’s performance. They identified turbidity as a main parameter influencing the feedwater characteristics. Utilizing UF reduced turbidity and showed that the levels of turbidity did not affect the seawater RO–PRO hybrid system’s performance considerably [71]. A comparative analysis of several pretreatment methods was performed by Ju et al. [72] at both bench and pilot scales. The bench-scale tests were cartridge filtration, MF, UF, NF, activated filter media, and granular activated carbon. In contrast, the pilot-scale ones focused on UF exclusively. The findings of the bench scale research showed that when coagulation was coupled with filtration, turbidity and the levels of total organic carbon were significantly decreased. However, pretreatment with cartridge filtration, MF, or UF alone lead to only slight reductions. Activated filter media, granular activated carbon, and NF exhibited significant influence in reducing turbidity and total organic carbon. On the other hand, the results of the pilot scale revealed a considerable effect of UF in turbidity but a slight reduction in other parameters. These findings highlighted the importance of choosing appropriate pretreatment methods for optimizing the quality of feedwater for PRO applications [72].

**Figure 4:**
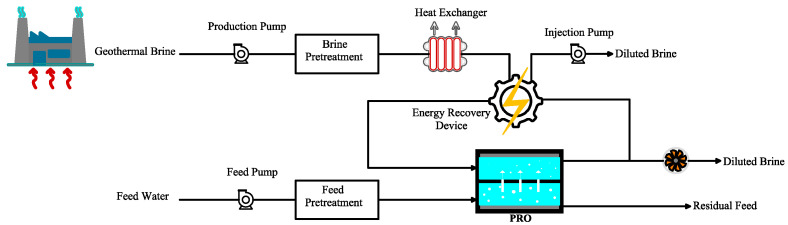
An integrated PRO with a geothermal heat plant system to provide electricity and heating (adapted from Madsen et al. [73]).

In another pilot study, Madsen et al. [73] evaluated various pretreatment methods by integrating geothermal heat and osmotic energy (Figure 4). The pretreatments included ion exchange, UF, anti-scalants, and nanofiltration. Among the tested methods, NF showed the best performance, leading to sustained 24 h energy production. They also demonstrated that it is necessary to apply tailored pretreatments for each feedwater due to their specific characteristics [73].

### 5.2. PRO Integrated with NF and RO

**Figure 5:**
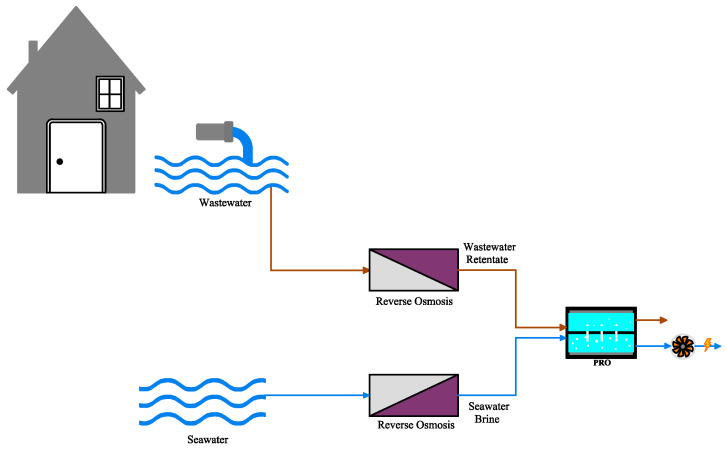
An integrated PRO with a reverse osmosis process using wastewater retentate and seawater desalination brine as feed and draw solutions, respectively (adapted from Wan et al. [74]).

Wan et al. [74] used UF and NF technologies for pretreatment of NEWater that is domestic waste in Singapore and considered as feed solution in the PRO plant (Figure 5). They used seawater brine from the first stage of RO as the draw solution during the PRO process. The hollow fiber thin-film composite membrane was utilized in the PRO system. Consequently, by pretreatment, the membrane performance was enhanced in the PRO process, and the fouling problem of feed solution was mitigated; seawater that had a higher salinity than the standard level was diluted and became safe for discharge, and the amount of energy during the seawater desalination in the RO system was recovered by PRO [74].

**Figure 6:**
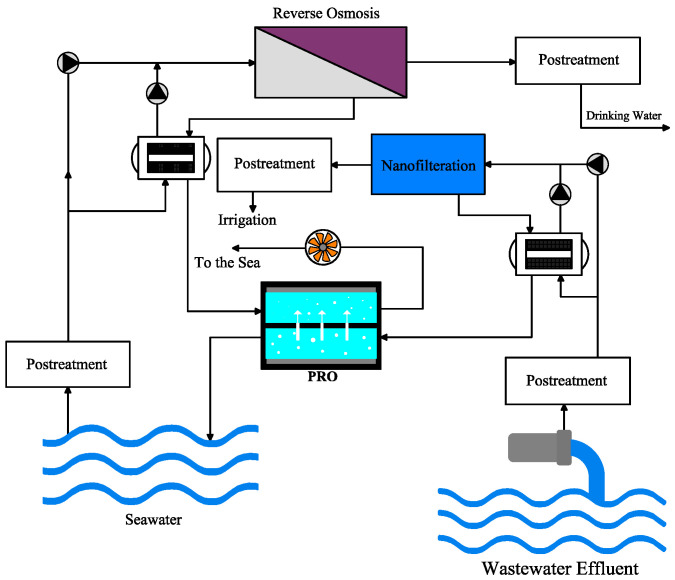
An integrated PRO process with NF and RO (adapted from Touati et al. [75]).

Touati et al. [75] proposed a RO–PRO-NF hybrid system to enhance energy and water management. As shown in Figure 6, the process starts with the pretreatment of wastewater effluent by NF, with the resulting permeate serving as the feed for PRO. Concurrently, seawater went through several pretreatments, involving coagulation–flocculation, sand filtration, cartridge filtration, and chemical treatments, before entering the RO. The concentrated brine generated by RO was used as DS for PRO. Wastewater pretreatment included chemical treatment, coagulation–flocculation, MF, and UF. This hybrid system exhibited the potential for hybridized treatment of wastewater, desalination of seawater, and production of energy, aligning with the food–water–energy nexus. Treated water from the system is appropriate for irrigation, further highlighting its multifaceted utility. An energetic assessment of the process revealed an energy output of 0.38 kWh/m^3^, and a techno-economic evaluation indicated that the system is economically viable under current PRO membrane performance levels, with a membrane price of $5/m^2^. However, higher costs of the membrane exceeding $15/m^2^ would necessitate considerable improvements of the performance for economic sustainability [75].

### 5.3. PRO Integrated with RO

Several researchers have explored the integration of PRO with RO to reduce the consumption of energy and to mitigate the discharge of brine into environments. Among other hybridizing configurations, the RO process is especially beneficial due to its capability to address environmental concerns stemming from the direct disposal of concentrated brine into the ocean. The RO desalination plant has two streams, including the permeate with low salinity that could be used as feed solution in PRO system and the RO brine with high salinity that can be used as draw solution during the PRO process. Moreover, using brine with high concentration as DS in PRO can improve the efficiency of power generation. In such hybrid systems, low-salinity water sources, like wastewater effluents, are merged strategically within the PRO with the concentrated brine stream generated by RO. This brine is used to recover energy by PRO instead of discharge into the environment. Accordingly, PRO functions through recovering energy from the brine and producing additional power using impaired sources of water. In addition, RO–PRO provides environmental advantages by diluting the brine came from RO with wastewater effluent before discharging, thereafter mitigating its environmental impact [18]. Sharqawy et al. [76] reported that RO–PRO can decrease the input power of RO by 38% [25].

The economic performance of another RO–PRO system was assessed by Sim et al. [77], and a total capital cost reduction of 8.7–20% was reported compared to standalone RO. This outcome emphasized the potential of RO–PRO for improving the efficiency of energy and for reducing the overall investment costs, rendering it a more economic option for desalination processes [77]. Kim et al. [78] performed a comparative assessment of four different designs for RO–PRO by a validated RO and a modified PRO model. The results showed the significant sensitivity of the performance of the hybrid system to costs of energy, especially in designs serving seawater as FS for RO. Furthermore, the research revealed that the required power input for such systems could be reduced, especially if the system configuration combined staged operations. As a result, this underscored the significance of considering energy costs and the size of the system for optimizing RO–PRO feasibility and efficiency [78]. Prante et al. [79] conducted a thermodynamic study and analyzed the RO–PRO hybrid system performance. It was found that net specific energy consumption of 1.20 kWh/m^3^ was considerably less than that of 2 kWh/m^3^ needed for standalone RO, representing a notable 40% decrease in consumption of energy, further highlighting the potential of RO–PRO to improve the efficiency of energy in desalination processes [79]. In 2014, Achilli et al. [80] designed and tested a pilot-scale RO–PRO system to analyze its feasibility for desalinating seawater and recovery of energy. The system combined three spiral-wound RO membranes and a spiral-wound TFC PRO membrane. Filtered municipal tap water and synthetic seawater were utilized as FS for PRO and RO, respectively. The research demonstrated several benefits of RO–PRO through reducing the consumption of energy via energy generation by PRO while diluting the RO brine back to seawater concentration. It was proven that RO brine was an effective DS due to its availability as a residual from existing systems with low-cost, its compatibility with the PRO after being pretreated by RO, and the absence of important foulants. The consumption of energy of RO was reduced from 3.82 kWh/m^3^ to 2.0 kWh/m^3^, and a power density of 1.1–2.3 W/m^2^ was achieved by PRO, representing the potential of energy recovery [80].

Altaee et al. [81] proposed a hybrid RO–PRO system for analyzing the effects of FS and DS concentrations on performance of the system, revealing that increasing the salinity of FS decreased the rate of permeate due to a decreased osmotic pressure difference across the membrane. In contrast, increasing the rate of DS improved the rate of permeate through creating a higher osmotic pressure gradient, while increasing the rate of FS had a slight influence on the rate of permeate. Consequently, the study highlighted the need to balance the advantages of higher rates of DS against their associated prices and potential performance challenges, underscoring the significance of optimizing operating conditions [81]. In another research study [82], the feasibility RO–PRO was examined, and the findings indicated that the feasibility of the hybrid system was enhanced with a lower rate of RO water recovery and a higher ratio of PRO FS flow rate to the combined rate of PRO FS and DS. In addition, obtaining optimal flux coupling numbers at lower dimensionless rates of water permeation for the same water recovery by RO needed a higher applied pressure but a reduced area of the membrane. Although the study offered valuable insights into the operational parameters that affected RO–PRO performance, it did not consider the impacts of CP or RSF, which are critical factors affecting the performance of membrane [82].

Wan et al. [83] developed a model for evaluating RO–PRO performance, reporting a specific energy consumption of 1.14 kWh/m^3^. The researchers also compared the efficiency of energy of a standalone RO with pressure exchangers to that of a RO–PRO hybrid system with the same mechanisms for recovering pressure. The results showed a 40–50% reduction in specific energy consumption for RO–PRO, depending on the recovery rate of desalinated water, further highlighting the potential for energy saving by integrating PRO with RO [83].

**Figure 7:**
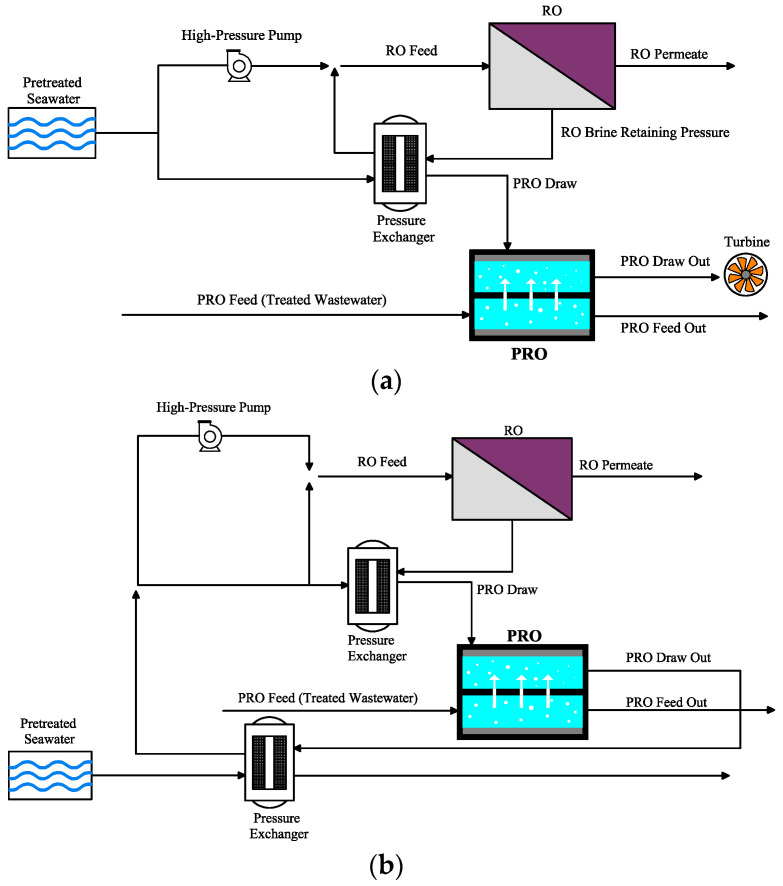
Schematic design of RO–PRO with (**a**) a turbine-based system and (**b**) an energy circulation system (adapted from Lee et al. [71]).

Altaee et al. [84] studied a RO–PRO hybrid system to mitigate RO brine rejection effectively, improving the desalination recovery rate by 18% with regard to the utilized salinity of the seawater. A PRO power density of 28 W/m^2^ was achieved with an RO recovery rate of 46% using 45 g/L seawater as the FS and 5 mol/L NaCl as the DS, emphasizing the potential of the hybrid configuration to enhance the efficiency of desalination while utilizing the contained energy in the RO brine for generation power [84]. In another study [85], a simplified assessing model was used to investigate the operating benefit of the RO–PRO system and demonstrated that hybridizing PRO with RO increased the recovery rate of RO, decreased specific energy consumption of desalination by 35%, and improved operating profit by 100%. It was indicated that the operating profit is affected by water and electricity fees, but the optimal operating conditions were identified by the ratio of these costs. Despite that the hybrid system offered substantial energy savings and profitability enhancements, it required additional capital investments in membranes, pumps, and energy recovery devices [85]. Li [86] analyzed the consumption of energy for a two-stage RO coupled with PRO by estimating their specific energy consumption based on rates of water recovery. The study showed that RO–PRO exhibited a higher consumption of energy, with a 50% increase in specific energy consumption in comparison with a 40% increase for the two-stage RO process [86]. Another RO–PRO system was studied with a focus on consumption of energy and changes in effluent properties. Two novel hybridizing configurations were suggested and assessed (Figure 7). It was reported that both designs reduced energy consumption of RO by 12–18%, depending on the recovery ratios, emphasizing the potential of hybrid system to enhance the efficiency of energy [87].

In another research study [71], two energy recovery designs of RO–PRO hybrid systems were proposed, including turbine-based and energy circulation systems. Turbine-based systems generate electricity through the rotation of turbines, providing flexibility for various applications, whereas an energy circulation system transfers energy directly into the seawater feed via a pressure exchanger, which reduces the energy demand of the RO process. The energy circulation system is typically preferred in pilot-scale systems because of the superior efficiency of pressure exchangers over turbines, making it the most practical application of PRO. However, the turbine-based system should not be overlooked since it enables PRO to perform as an independent source of renewable energy, expanding its application. Therefore, the pilot-scale RO–PRO demonstrated the viability of PRO in decreasing the specific energy consumption and improving energy recovery in desalination processes and addressing environmental issues [71].

For treatment of water with very high salinity, double-RO desalination is suggested to generate a feed solution with lower salinity, to be used in the PRO system. In a study, they used dual-stage PRO with RO brine to generate a high amount of renewable energy. Dymola software was used, and it made two streams including two RO systems with PRO and a second one, which was two RO systems with a double PRO system. The salinities of the seawater were 40 and 45 g/L. The savings of energy for two RO with a double PRO system was 17.5% at 47% recovery of water for 45 g/L of seawater salinity which was more energy efficient in comparison with two RO systems. Their results showed the high efficiency of combining two RO systems with double PRO to decrease the environmental impacts and consumption of energy during the desalination process [88]. Japan’s Mega-ton Water System, which combined seawater RO with PRO, was the most prominent example of an integrated PRO hybrid process. A hollow fiber membrane was used with the brine of seawater RO as the draw solution and regional wastewater as the feed solution. Power around 13 W/m^2^ was achieved when a hydraulic pressure of 30 bar applied to the system, and approximately 10 to 30% energy could be saved during the proposed hybrid system [89].

### 5.4. PRO Integrated with FO

**Figure 8:**
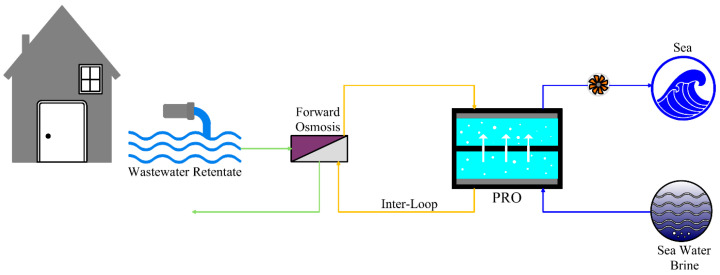
An integrated PRO with a forward osmosis process using wastewater retentate and seawater brine as feed and draw solutions, respectively (adapted from Cheng et al. [90]).

An innovative FO-PRO hybrid system aimed to generate sustainable osmotic power, serving real wastewater effluent from a municipal water recycling plant as FS (Figure 8). The FO process extracted clean water from wastewater effluent to the inter-loop solution, which was then used as a clean feed for the PRO. This hybrid system harnessed the inherent benefits of FO, such as low propensity to fouling, easy membrane cleaning, and less external energy needs, rendering FO-PRO as a promising option for sustainable power generation. The outcomes of the study indicated that FO removed salts and foulants from wastewater effluent effectively, yielding a clean inter-loop solution with trace amounts of silica and inorganic ions. However, FO membranes exhibited a mild decline of flux due to CP and RSF. The active layer of the FO membrane prevented fouling from penetrating into the support layer, with fouling confined to the surface of the membrane. Silica scaling and inorganic fouling were determined, but hydrodynamic shear forces reduced their effect on transport of water. The DS of PRO was diluted back to seawater, facilitating easy recycling or disposal, and a power density exceeding the commercial benchmark of 5 W/m^2^ was achieved. This hybrid system outperformed standalone PRO systems due to less fouling from the use of wastewater effluent as a FS. The FO pretreatment decreased maintenance prices and prolonged the lifespan of the membrane, which improved the overall cost effectiveness [90].

### 5.5. PRO Integrated with FO and RO

**Figure 9:**
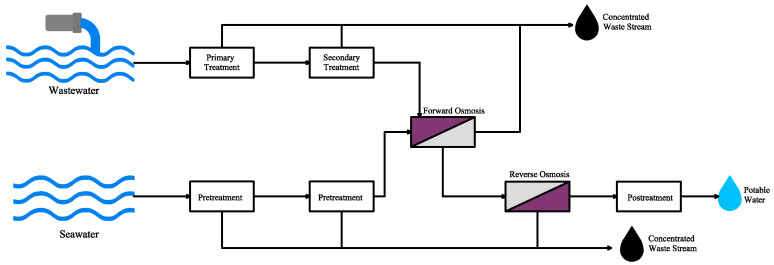
An integrated PRO with a forward and reverse osmosis processes (adapted from Cath et al. [91]).

In simple words, forward osmosis (FO) is used for generating safe draw solutions, RO for water purification, and PRO for harvesting renewable energy. Sometimes, integrating wastewater and seawater RO brine is referred to as a FO-RO hybrid system (Figure 9). In the first step of the FO-RO system, water is moved from the solution with low salinity to the solution with higher salinity (draw solution) due to osmotic pressure difference between these two solutions in FO process. A study investigated some advantages of the FO-RO hybrid system compared with a single RO desalination plant. Benefits include the fact that less energy is needed for seawater RO desalination due to lower pressure that is required because of dilution during osmotic process. It is possible to reuse the wastewater and decrease the fouling problem because of the contaminant’s dilution and lower utilized pressure [91]. Furthermore, researchers found that the integration of the FO-RO processes with PRO has many advantages, including higher power generation and mitigating fouling problems by treatment of wastewater [92].

Furthermore, researchers found that integration of the FO-RO processes with PRO has many advantages including higher power generation and mitigation of fouling problems by treatment of wastewater [92].

### 5.6. PRO Integrated with MD

**Figure 10:**
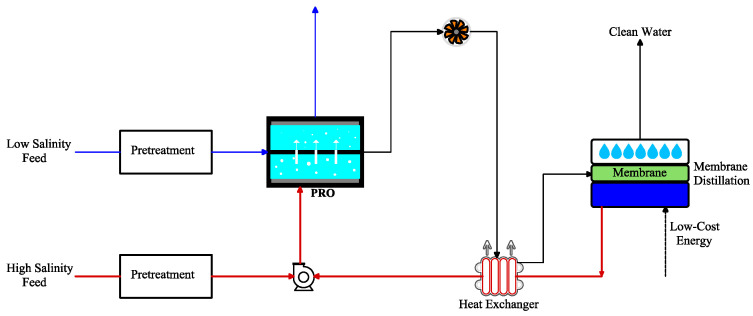
Integrated PRO with membrane distillation (adapted from Han et al. [94]).

The PRO-MD hybrid system is a novel approach to produce energy and clean water at the same time, employing waste streams. In PRO-MD (Figure 10), MD concentrates and heats the seawater brine from RO to produce clean water, along with generating concentrated a DS with a high temperature for PRO. By harnessing low-grade heat sources for both freshwater and energy generation, the integrated design improves the efficiency of the overall system by merging two processes in a single module, and it further reduces the volume of wastewater, and it mitigates environmental impacts. Lin et al. [93] studied a closed-loop MD-PRO to enhance water recovery and energy generation. In this hybrid system, MD produced concentrated and purified waters using thermal separation, whereas PRO converted the salinity gradient between solutions with high and low concentrations into electricity. The study showed that the practical efficiency of energy decreased in comparison with that of theoretical value due to challenges in mass and heat transfer kinetics [93]. Another PRO-MD system was suggested by Han et al. [94] in which the diluted DS from PRO was entered to MD as feed, resulting in minimal membrane fouling with high rates of water recovery and osmotic power. The obtained power densities were 31 W/m^2^ and 9.3 W/m^2^ for water and wastewater, respectively, as a FS and a concentrated 2 M NaCl as a DS. The potable water flux in MD was 30–60 L m^−2^ h^−1^, utilizing a feed solution with 40–60 °C temperatures [94].

Lee et al. [95] hybridized PRO with a multi-stage vacuum MD system to enhance the recovery of water and energy. In this system, the concentrated brine from the multi-stage vacuum MD was fed into PRO as a DS, and on the contrary, distilled water was produced as the FS. The flows were continuedly recycled in the process for producing brine and distilled water. The study found an inverse correlation between the ratio of the recycled flow and the amount of produced distillate at a fixed rate of feed flow. The concentration of brine was 1.9 M, with a feed flow rate of 50 g/s and a 90% recycle ratio. In PRO, serving river water as the FS at an applied pressure of 13 bar led to a power density of 9.7 W/m^2^. This integration highlighted the flexibility of PRO-MD, especially for water applications with low salinity [95]. In 2017, theoretical research on PRO coupled with direct contact MD was performed by Park et al. [96] to predict the production of water and power, and the results were compared with a standalone PRO and direct contact MD systems. In the suggested hybrid system, a hydrophobic membrane separated a cold DS and a hot FS. The results revealed that the integrated system decreased the overall consumption of energy by 0.1738 kWh/m^3^ because of the power produced by PRO. However, this enhancement in efficiency of energy came at the cost of decreased water flux in comparison with the standalone systems [96].

### 5.7. PRO Integrated with MD and RO

Kim et al. [97] conducted a study to investigate the performance of the RO-MD-PRO hybrid system by introducing the concept of a brine division ratio as a main parameter to optimize the efficiency of the process. It was concluded that the proposed hybrid system not only decreased specific energy consumption but also reduced the environmental footprint, especially the marine impacts. A 1.6 kWh/m^3^ of specific energy consumption was obtained at a brine division ratio of 1.0, representing a 17% decline associated with the standalone RO [97]. Choi et al. [98] assessed the performance and economics of RO-MD-PRO via a theoretical analysis [98]. Chae et al. [99] proposed a novel dimensionless performance index for comparing the efficiency of energy between RO–PRO and RO-MD-PRO hybrid systems after running several simulations. It was revealed that RO-MD-PRO had a higher efficiency of energy compared to that of RO–PRO [99]. Table 3 demonstrates some of the studies that worked on the integration of other processes with the PRO system.

## 6. Multidisciplinary Advances in PRO Pretreatment Integration

### 6.1. PRO Integrated with Membrane Bioreactor

**Figure 11:**
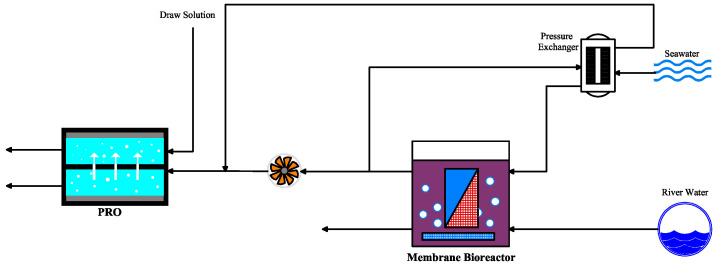
Integrated PRO with a membrane bioreactor system (adapted from Chung et al. [100]).

Tai-Shung et al. [100] conducted research on integrating RPO with a membrane bioreactor system to generate power with seawater desalination (Figure 11). In this hybrid system, seawater was pressurized via a pressure exchanger and served as the DS for PRO, while wastewater pretreated with membrane bioreactor was used as the FS. A portion of the diluted DS was directed toward the pressure exchanger for pressurizing incoming seawater, while the remaining portion was directed to a turbine to generate power. The study demonstrated simultaneous power production and contaminant removal from wastewater despite limitations with severe fouling [100].

### 6.2. PRO Integrated with RO and Solar Energy

An integrated PRO system with RO and solar energy was proposed to optimize the desalination of seawater and power generation. The hybrid system pressurized seawater via a pump and an energy recovery device before feeding it through RO, which produced freshwater and concentrated brine. The concentrated brine was reused in the energy recover device to improve the efficiency of energy, and PRO generated electricity by harnessing the salinity gradient between the low-concentration FS and brine. The source of energy for the proposed hybrid system involved solar energy, leveraged during the day via photovoltaics and the salinity-gradient energy from PRO, offering electricity to RO. Therefore, the system utilized both photovoltaics and PRO throughout the day and relied solely on PRO during the night, ensuring uninterrupted function. This hybrid system provided several profits, including sustainable energy incorporation, continuous generation of power, application of non-conventional water sources, and scalability for deployment in remote areas. This innovative hybrid system offered an environmentally friendly and energy-efficient option to conventional desalination technologies, addressing the challenges associated with water and energy in regions with high solar irritation and resources of seawater. Consequently, solar energy had a vital role in the proposed hybrid system by offering a sustainable and renewable energy source to power the process of desalination. The overall efficiency and productivity of the system improved through a significant increase in water production, reducing dependency on fossil fuels, and facilitating synergy between renewable and osmotic energies. This makes this integrated system a viable solution for seawater desalination, environmentally and economically [101].

**Figure 12:**
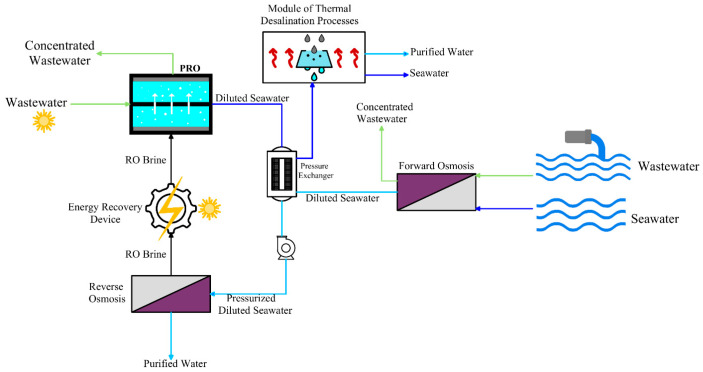
Thermal desalination process integrated with temperature-enhanced PRPO (adapted from Wang et al. [102]).

Integrating PRO with solar energy and thermal desalination processes represents a promising hybrid alternative using a DS with high temperature and low salinity (Figure 12). This innovative design improved the efficiency of salinity gradient energy, reduced reliance on fossil fuels, and optimized energy utilization across desalination stages. In the proposed hybrid system, solar energy performed two critical roles involving the increase in salinity gradient energy efficiency in PRO by raising operating temperatures and flow rates and preheating the FS, which reduced the required thermal energy in the subsequent thermal desalination stage. The hybrid system also incorporated FO to dilute high-salinity FSs and reduce energy needs and further enhance the compatibility of operating pressures between PRO-RO. The salinity of the FS decreased by diluting seawater in FO, contributing to lower consumption of energy in RO. Diluted DS from PRO was fed into thermal desalination processes, such as multi-stage flashing or multi-effect distillation. These systems benefited from the DS with high temperature and low salinity, reducing scaling, minimizing consumption of thermal energy, and lowering the discharge of brine, leading to zero liquid discharge. Solar energy was derived from the hybrid system through solar linear Fresnel collectors and concentrated solar power systems. Solar linear Fresnel collectors were selected due to their cost efficiency, lower land requirements, and ease of installation, whereas concentrated solar power systems fulfilled the system’s thermal energy needs significantly. The integration of solar energy in this hybrid system as a thermal source led to an increase in operating temperature and significantly improved PRO performance. Higher operating temperatures enhanced water flux and optimal operating pressure, resulting in a 1.82-fold increase in power density. Using high-temperature and low-salinity discharge from this integrated system reduced the thermal input required for subsequent desalination processes, thereby improving the overall efficiency of the system. Consequently, the hybrid system exhibited a sustainable and efficient alternative for seawater desalination and energy generation [102].

### 6.3. PRO Integrated with RO and Wind Energy

**Figure 13:**
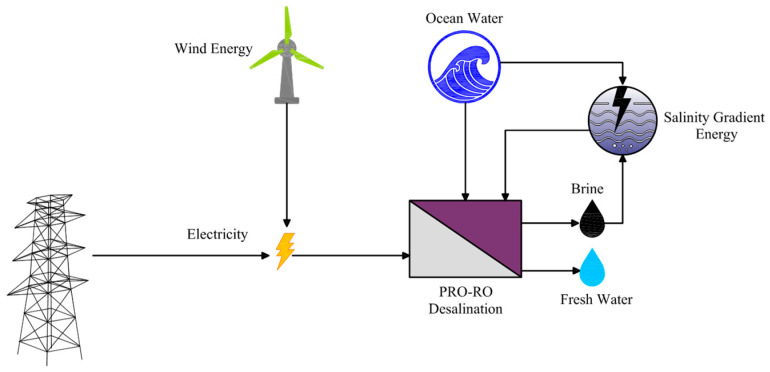
An integrated PRO with RO and wind energy (adapted from Okampo et al. [57]).

PRO was integrated with RO and offshore wind energy to provide a sustainable, cost-efficient option for freshwater production and energy generation (Figure 13). In this hybrid system, the utilization of PRO reduced the pressure required for RO and recycling brine within the system eliminated disposal fees and environmental concerns while offshore wind power minimized carbon emissions to 193,323 kgCO_2-e_ and energy prices. Economic analysis highlighted the viability of the hybrid system, with a $1.11/kW levelized cost of energy and a $0.13/m^3^ of water cost demonstrating a substantial enhancement in cost efficiency in comparison with standalone RO systems. The proposed hybrid system addressed freshwater scarcity and environmental risks, providing a reliable option for areas with abundant wind and ocean resources. The integration of wind energy into the hybrid system resulted in significant environmental and economic advantages via facilitating brine management and reducing the related expenses and the environmental burden of brine disposal. Ultimately, these outcomes highlighted the potential of the proposed hybrid system to advance desalination practices by economic viability, environmental sustainability, and energy efficiency [57].

## 7. Concluding Remarks

Water and renewable shortage are now global issues that endanger both the environment and human health due to the trend of population growth and industrialization. A lot of industrial activities release poorly treated wastewater, which frequently causes direct or indirect environmental contamination. Different membrane-based technologies have been utilized for recovery and reuse of water by treating oily wastewater, water containing organic, inorganic, and heavy metals and other materials [59,103]. One possible method to generate renewable energy by using the natural salinity gradient is the PRO process. By preparing a suitable membrane, it is possible to design a hybrid system with higher efficiency to mitigate environmental impacts and generate electricity. In recent years, researchers have investigated various PRO configurations, multi-stage or hybridized process designs, and ways to increase overall process efficiency while lowering energy consumption and operating costs. Combining membrane-based technologies can recover heat and natural resources in addition to generating energy. Pretreatment techniques are also thought to extend membrane aging, minimize the requirement for chemical and physical cleaning and module replacement, and decrease PRO membrane fouling. For example, the RO-NF-PRO hybrid process is a remarkable system that combines various membrane processes to concurrently achieve some goals including pretreatment of the feed, preconcentration of the draw solution, and energy generation. Moreover, many studies investigated novel nanomaterials to increase the membrane efficiency by enhancing their water permeability, selectivity, and stability. However, the cost of material synthesis and process optimization for efficient and reliable incorporation and modification in membrane-based technologies are some of these techniques’ drawbacks that need to be considered. Membrane modules need to be made in a way that minimizes pressure drop, minimizes membrane weak points, prevents deformation, and increases their lifespan to work longer periods of time [104].

Depending on the characteristics of the wastewater, other pretreatment conditions may also be considered, and based on simulation and experimental studies hybrid processes can be developed in the future. The future of PRO research must also include energy analysis and environmental evaluations to assess system sustainability, economic viability, and efficiency. Furthermore, life cycle assessments are necessary to assess the economic and environmental trade-offs of these hybrid systems. As a result, further studies on the design and large-scale installation of a hybrid system are essential for wastewater treatment and sustainable energy generation.

## Figures and Tables

**Table 1:** Summary of different materials used for membrane modification.

Sample	Type of Material	Type of Membrane	Modification Method	Membrane Thickness	Temperature °C	FS	DS	Power Density (W/m^2^)	Improvements	Ref.
TFC-(Na-CQD-9)-1	Functionalized Carbon Quantum Dots	Hollow fiber	Active layer	-	Room temperature	Deionized water	1.0 M NaCl	34.20	Increase in hydrophilicity, water flux, and power density	Gai et al. [36]
PP-0.002 (PP-SO_3_H-loaded membrane)	Sulfonate-functionalized porous polymer (PP-SO_3_H)	Hollow fiber	Active layer	324 ± 12 μm	Ambient temperature	Deionized water	1.0 M NaCl	14.6	A significant increase in water flux to 46.3 L m^−2^ h^−1^, compared to 31.4 L m^−2^ h^−1^ for unmodified membrane	Gonzalez et al. [37]
PDA@PSf-1 h	Polydopamine-modified polysulfone substrate	Flat sheet	Active layer	100 μm	Ambient temperature	Deionized water	2.0 M NaCl	N/A	PDA coating enhanced water flux from 7.5 L m^−2^ h^−1^ to 24 L m^−2^ h^−1^ and improved salt rejection	Han et al. [38]
TFC4	Polyamide active layer modified by triaminopyrimidine monomer and a PDA/GO interlayer	Commercial flat sheet	Active layer	-	25–65	Deionized water	1.0 M NaCl	N/A	Highest thermal stability, with less variation in water flux and reverse salt flux under elevated temperatures (65 °C).	Karami et al. [40]
M-0.05	Biowaste-derived nitrogen-doped carbon quantum dots	Flat sheet	Active layer	100 μm	25 ± 1	Deionized water	1.0 M NaCl	N/A	Achieved the highest water flux (35.3 L m^−2^ h^−1^) with minimal reverse solute flux (0.154 g/L). Structure parameters decreased to 174 μm, reduced ICP.	Mazhari et al. [41]
M-0.1	Zeolite	Flat sheet	Support layer	200 μm	Room temperature	N/A	N/A	N/A	Modified NF membrane with zeolite, enhanced water permeability, and excellent divalent salt rejection	Mohammad et al. [44]
TFN-0.25GO	GO	Flat sheet	Support layer	200 μm	Room temperature	Deionized water	1.0 M NaCl	8.36	Enhanced water flux to 30.95 L m^−2^ h^−1^ and its structural parameter reduced.	Idris et al. [43]
TFN0.4	Zeolite	Flat sheet	Support layer	100 μm	Room temperature	10 mM NaCl	2.0 M NaCl	N/A	Analyzed in FO and PRO mode; water flux increased by approximately 40% compared to TFC with acceptable salt rejection.	Salehi et al. [45]
PDA/GO0.5	PAN/PPSU substrate and PDA/GO coating	Flat sheet	Support layer	110 ± 5 μm	24	Aerobically treated palm oil mill effluent	4.0 M MgCl_2_	N/A	Increase in water flux: 67% higher in FO mode and 41% higher in PRO mode compared to uncoated TFC. High color removal rates up to 97.37%.	Abdullah et al. [46]
T-GO/HNT4	HNTs and GO	Flat sheet	Support layer	~100 μm	23	Deionized water	1.0 M NaCl	16.7	High power density and mechanical strength achieved. Only 17% decline during fouling tests in comparison with 57% for commercial membrane.	Lim et al. [47]
THF-GO-0.2	GO	Hollow fiber	Support layer	~190 μm	23 ± 1	Deionized water	1.0 M NaCl	14.6	Achieved the highest water flux of 43.74 L m^−2^ h^−1^ and the lowest specific reverse salt flux. Enhanced water permeability and mechanical stability.	Park et al. [48]

**Table 2:** Summary of modified TFC membrane with antifouling properties.

Sample	Type of Membrane	Type of Material	Membrane Thickness	FS	DS	Benefits	Ref.
1	Flat sheet FO and PRO	Zwitterionic arginine	100 μm	Oily wastewater	1 M NaCl	Enhanced the hydrophilicity combined with positive charge of membrane surface, which caused high antifouling and antibacterial properties of membrane.	Chen et al. [52]
2	Flat sheet FO	MoS_2_-Ag	120 μm	Synthetic wastewater	2 M NaCl	Decrease in contact angle from 79° to 52° (from TFC to TFN, respectively), which significantly enhanced membrane resistance towards alginate fouling.	Kim et al. [53]
3	Flat sheet FO and PRO	GO	-	Synthetic wastewater	1.6 M NaCl	Controlled biofouling by increasing hydrophilicity and decreasing water flux and ICP	Li et al. [54]
4	Commercial flat sheet	ND	-	Synthetic wastewater	2 M NaCl	ND nanoparticles decreased the electrostatic interface between the membrane and organic foulants and additionally improved the antibacterial properties.	Karami et al. [55]

**Table 3:** Summary of combined processes with PRO.

System	FS	DS	Membrane	Power (W/m^2^)	Key Findings	Ref.
UF-PRO	Wastewater retentate pH: 7.44 TOC: 44.05 ppm	Seawater brine pH: 7.30 TOC: 66.17 ppm	Hollow fiber	6.6	Without pretreatment: Power density decreased to 4.6 W/m^2^ because of fouling. UF pretreatment increased power density to 6.6 W/m^2^.	Wan et al. [74]
UF-PRO	Wastewater retentate with high levels of Ca^2+^, Mg^2+^, SO_4_^2−^, and PO_4_^3−^	Seawater brine NaCl: 0.8 M	Hollow fiber	2.92	UF pretreatment was identified to be ineffective in reducing fouling.	Yang et al. [70]
UF-PRO	Wastewater	Seawater brine	Hollow fiber	13.5	The integration of UF was effective in maintaining stable operation over a 14-day period.	Sakai et al. [67]
NF-PRO	Wastewater retentate	Seawater brine	Hollow fiber	8.9	Without pretreatment: Power density decreased to 4.6 W/m^2^ because of fouling. NF pretreatment increased power density to 8.9 W/m^2^.	Wan et al. [74]
NF-PRO	Wastewater retentate with high levels of Ca^2+^, Mg^2+^, SO_4_^2−^, and PO_4_^3−^	Seawater brine NaCl: 0.8 M	Hollow fiber	7.3	NF showed effective removal of foulants and scaling agents	Yang et al. [70]
NF-PRO	Wastewater	NaCl: 5 M	Hollow fiber	13.5	Key contributors to fouling were organic substances, inorganic calcium salts, and silica. NF showed effective removal of multivalent ions and organic compounds under low pressure and substantially mitigated fouling, leading to a three-fold increase in water flux.	Chen et al. [69]
RO–PRO	Wastewater effluent	Seawater brine	Hollow fiber	~13	Maximum power density was achieved by 10-inch hollow fiber modules.	Tanioka et al. [89]
RO–PRO	Wastewater retentate with high levels of Ca^2+^, Mg^2+^, SO_4_^2−^, and PO_4_^3−^	Seawater brine NaCl: 0.8 M	Hollow fiber	8.4	RO showed effective removal of foulants and scaling agents	Yang et al. [70]
RO–PRO	Freshwater	Seawater	FilmTec BW30	10	The development of biofilm and intensity of fouling were directly related to flux and CP. Biofouling was increased substantially at higher fluxes.	Prante et al. [79]
FO-PRO	Wastewater NaCl: 0.011 M	Diluted seawater brine NaCl: 1.2 M	Hollow fiber	>5	A power density higher than 5 W/m^2^ was obtained with less fouling and sustainable operation.	Cheng et al. [90]
MD-PRO	Deionized water Wastewater 1: TOC: 500 ppm TDS: 88,000 ppm Conductivity: 118 mS/cm Wastewater 2: TOC:100 ppm TDS: 93,000 ppm Conductivity: 124 mS/cm Wastewater 3: TOC: 100 ppm TDS = 314,000 ppm Conductivity: 252 mS/cm	NaCl	Flat sheet Hollow fiber	31 9.3	MF removed 90% of TOC from wastewater 1. MF removed 62% of TOC from wastewater 2. MF removed 73% of TOC from wastewater 3.	Choi et al. [98]
MD-PRO	River water	NaCl	CA TFC	9.7	CTA membranes demonstrated superior resistance to fouling by retaining 94% of initial flux while TFC membranes retained only 41–76%	Lee et al. [95]

## Data Availability

No new data were created or analyzed in this study. Data sharing is not applicable to this article.
